# HIV and infant feeding counselling: challenges faced by nurse-counsellors in northern Tanzania

**DOI:** 10.1186/1478-4491-5-18

**Published:** 2007-07-24

**Authors:** Sebalda C Leshabari, Astrid Blystad, Marina de Paoli, Karen M Moland

**Affiliations:** 1 School of Nursing, Muhimbili University College of Health Sciences, Dar es Salaam, Tanzania; 2 Centre for International Health, University of Bergen, Norway; 3 Bergen University College, Norway; 4 Department of Public Health and Primary Health Care, University of Bergen, Norway; 5 Fafo Institute of Applied International Studies (AIS), Norway

## Abstract

**Background:**

Infant feeding is a subject of worry in prevention of mother to child transmission (pMTCT) programmes in settings where breastfeeding is normative. Nurse-counsellors, expected to counsel HIV-positive women on safer infant feeding methods as defined in national/international guidelines, are faced with a number of challenges. This study aims to explore the experiences and situated concerns of nurses working as infant feeding counsellors to HIV-positive mothers enrolled in pMTCT programmes in the Kilimanjaro region, northern Tanzania.

**Methods:**

A qualitative study was conducted using in-depth interviews and focus group discussions (FGDs) with 25 nurse-counsellors at four pMTCT sites. Interviews were handwritten and FGDs were tape-recorded and transcribed, and the programme Open Code assisted in sorting and structuring the data. Analysis was performed using 'content analysis.'

**Results:**

The findings revealed a high level of stress and frustration among the nurse-counsellors. They found themselves unable to give qualified and relevant advice to HIV-positive women on how best to feed their infants. They were confused regarding the appropriateness of the feeding options they were expected to advise HIV-positive women to employ, and perceived both exclusive breastfeeding and exclusive replacement feeding as culturally and socially unsuitable. However, most counsellors believed that formula feeding was the right way for an HIV-positive woman to feed her infant. They expressed a lack of confidence in their own knowledge of HIV and infant feeding, as well as in their own skills in assessing a woman's possibilities of adhering to a particular method of feeding. Moreover, the nurses were in general not comfortable in their newly gained role as counsellors and felt that it undermined the authority and trust traditionally vested in nursing as a knowledgeable and caring profession.

**Conclusion:**

The findings illuminate the immense burden placed on nurses in their role as infant feeding counsellors in pMTCT programmes and the urgent need to provide the training and support structure necessary to promote professional confidence and skills. The organisation of counselling services must to a larger extent take into account the local realities in which nurses construct their role as counsellors to HIV-positive childbearing women.

## Background

Infant feeding counselling based on international guidelines is considered a cornerstone in the prevention of mother-to-child transmission of HIV. Whereas perinatal anti-retroviral prophylaxis currently administered through standard pMTCT programmes in sub-Saharan Africa greatly reduces the transmission of HIV to the baby during labour and delivery, it does not reduce transmission during breastfeeding. Despite routine counselling on infant feeding, HIV-positive women enrolled in pMTCT programmes are commonly left desperately uncertain about how best to feed their infants. Exposed to pressures from family and friends, many end up feeding their infants in ways that may increase the risk of HIV transmission. In this context, the quality of the infant feeding counselling and the knowledge and practices of nurses providing the services have been called into question.

An increasing body of research documents the shortcomings of infant feeding counselling particularly in terms of counsellors' knowledge about pMTCT and counselling skills [1-4]. However, the experiences of counsellors have not been the focus of previous enquiry, and little is known about how the counsellors themselves perceive and experience their work in pMTCT programmes. With the aim of increasing our knowledge of the problems associated with the provision of infant feeding counselling, this study sets out to explore the experiences and situated concerns of nurses working as infant feeding counsellors to HIV-positive mothers enrolled in pMTCT programmes in the Kilimanjaro region, northern Tanzania.

Mother-to-child transmission of HIV (MTCT) represents a major threat to the gains in child health achieved during the last decades and represents a huge public health problem in HIV-affected populations, especially as it threatens breastfeeding [5]. It is estimated that in the absence of any intervention, 30–45% of infants born to HIV-positive mothers who breastfeed for 18–24 months will be infected with HIV either during pregnancy and birth or during the period of breastfeeding. Perhaps as much as 40% of these infections may occur during breastfeeding when this is extended for two or more years [6]. Partial and mixed feeding, in which breastfeeding is combined with other fluids or solids and fluids respectively, carries a higher risk of HIV infection than exclusive breastfeeding (breastfeeding only with no supplementation of any kind) [7-10]. In a study from Zimbabwe in 2005, Iliff and colleagues found that early mixed feeding was associated with a four-fold increased risk of postnatal HIV-1 transmission at six months compared to exclusive breastfeeding [9]. Exclusive breastfeeding, moreover, has protective properties and prevents common infections in babies [11].

In response to the risk of HIV transmission through breastfeeding, the current international guidelines for HIV and infant feeding state that "*when replacement feeding is acceptable, feasible, affordable, sustainable, and safe (AFASS), avoidance of all breastfeeding by HIV-positive mothers is recommended. Otherwise, exclusive breastfeeding is recommended during the first months of life*"[12]. The guidelines also state that HIV-positive mothers should receive individual counselling on the risks and benefits of the different infant feeding options including exclusive breastfeeding or exclusive replacement feeding with either animal modified milk or industrial infant formula. Furthermore, based on the principle of informed choice, women should be given the necessary guidance and support to enable them to choose the most appropriate option for their particular life situation while taking the AFASS criteria into account [12].

These guidelines gives details of infant feeding counselling in projects to prevent MTCT which routinely offer a standard package of voluntary counselling and testing (VCT), anti-retroviral prophylaxis and modified delivery services in addition to infant feeding counselling [13,14]. Nurses/midwives constitute the backbone of pMTCT programmes and represent the largest group of health workers available to counsel women on the recommended safer infant feeding practices in most African countries [15]. Holding a key role in service provision, close to the patient, and provided with accurate information on the risks and benefits of different feeding options, nurses are considered a group that is able to influence mothers' decisions on infant feeding and that can thus contribute to the reduction of postnatal transmission of HIV [16,17]. Advocates of exclusive breastfeeding have concluded that with formal training and supportive supervision, health workers can effectively increase rates of exclusive breastfeeding [18-20]

The experience from United Nations Children's Fund (UNICEF) pMTCT programmes' evaluation clearly shows that the infant feeding component is still weak [21]. A number of studies have documented that the quality of counselling on infant feeding remains unsatisfactory [1,2]. It has been documented that both counsellors and mothers are not sufficiently well informed about how to protect the infants from HIV transmission [2], and that counsellors are not always aware of the existence of current international guidelines on HIV and infant feeding [2,22]. In fact, not all pMTCT counsellors are trained in infant feeding counselling [21]. In addition to the documented breach in updated knowledge on HIV and infant feeding, the counsellors' practices as care providers have been heavily criticised [23,24]. Counsellors are frequently pressured for time and have too little insight into the mother's personal circumstances to offer appropriate comment and recommendations on the basis of the AFASS criteria [25]. After a mother makes her infant feeding choice the support available to assist her to practise her choice successfully is even more limited [1].

A study in South Africa which observed and interviewed counsellors about how they informed mothers about infant feeding found that the HIV-negative women had been informed about the advantages of exclusive breastfeeding, but only a minority of the HIV-positive women had been told about the risk of breast milk transmission when complementary food was added [1]. None of the mothers had been properly informed about the advantages and disadvantages of replacement feeding [1]. In a study of the differences between the international recommendations on breastfeeding and counselling messages of health workers in Malawi, Piwoz and colleagues found that misconceptions were common and that counsellors were strongly influenced by cultural beliefs about infant feeding [26].

To date only few studies have focused specifically on counsellors' perspectives in providing infant feeding counselling. A sub-study in a VCT efficacy study from sites in Kenya and Tanzania documented a high level of stress among the counsellors related to the emotional burden of dealing with issues closely associated with life and death as well as with heavy patient flow and a limited staff support system [27].

PMTCT efforts in Tanzania started in 2000 through pMTCT pilot sites and are currently being rolled out nationally. With an estimated HIV prevalence rate of 12% for antenatal women and a total vertical transmission rate of approximately 40%, an estimated 72,000 babies in Tanzania will become infected with HIV from their mothers per year [13]. Approximately 25,000 of these will be infected through breastfeeding [13]. The national infant feeding guidelines follow the international guidelines, and women are counselled to choose either (a) exclusive breastfeeding with early weaning at four to six months or at any time convenient in the individual woman's situation, or (b) replacement feeding with commercial infant formula, and/or (c) replacement/home-modified formula (cow's or goat's milk) when AFASS criteria can be met [13]. No free infant formula is provided as part of the programme.

The guidelines further explain that HIV-positive mothers who choose not to breastfeed should receive education and support on how to prepare and give their infant the replacement food. Mixed and partial breastfeeding is strongly discouraged. It is emphasised that the mother herself should make the final choice of feeding method and that whatever her choice, a counsellor should provide support to ensure optimal nutrition of mother and child [13]. It is also clearly stated that the counsellors in pMTCT programmes should be nurses/midwives who have undergone at least six weeks' training in counselling including VCT [13]. In spite of policy guidelines at the international and national level, infant feeding counselling remains a major challenge and a controversial issue in pMTCT in Tanzania [2].

A qualitative study in Moshi, Kilimanjaro region in 2000, investigating counsellors' infant feeding advice to HIV-positive women, concluded that infant feeding options were not accurately explained and that informed choice of infant feeding method, as recommended in the guidelines, was seriously compromised by inadequate information, directive counselling, lack of time, and lack of follow-up support [2]. Using this study as a point of departure, we have gone one step beyond investigating nurse-counsellors knowledge and practices to ask: *Why is the quality of counselling not good enough? *Situated at the centre of the pMTCT programme as service providers and at the same time being women exposed to the same risks as their clients, nurse-counsellors are invaluable sources of information. The aim of this study is to represent the perspectives of nurse-counsellors. The article seeks to explore nurse-counsellors' perceptions of the relevance of the infant feeding guidelines in the particular cultural and social setting of the Kilimanjaro region, northern Tanzania; the dilemmas facing nurse-counsellors in their everyday work; and their job satisfaction as counsellors in the pMTCT programme.

## Methods

### Study setting

This study was conducted at four pMTCT sites in Moshi Town, the administrative capital of the Kilimanjaro region in northern Tanzania. These four sites comprised the two largest health centres in Moshi town, the regional hospital and the referral hospital. The four sites are all characterised by heavy patient load and target both urban and rural populations. The catchment area includes the Moshi district, which has an estimated population of 144 336 people living in Moshi town and 402 431 people living in the surrounding rural areas [28]. The HIV prevalence rate in the antenatal population in Kilimanjaro region is estimated at 5.7% [29]. According to the latest National Demographic and Health Survey, 98% of all pregnant women in Kilimanjaro region attend antenatal clinic at least once during pregnancy, and female literacy rate is estimated at 91.6% [30]. The same survey report documents that 35% of the population of Tanzania have access to piped water, 13% to a protected well and 6% to a protected spring [30]. About 88% of Tanzanians use firewood as fuel for cooking and only 1% of the rural population have access to electricity [31].

The most dominant ethnic group in the study area is the Chagga who inhabits the slopes of Mt. Kilimanjaro. In the Kilimanjaro region prolonged breastfeeding and early supplementation with water, cow's milk and porridge is common [32]. All four study facilities provide services to both urban and rural populations. Three of the facilities offer mainstream HIV services, such as VCT, infant feeding counselling, and the treatment of opportunistic infections, but do not offer antiretroviral prophylactics. The fourth pMTCT site was at Kilimanjaro Christian Medical Centre (KCMC) which is one of the five national pilot pMTCT sites. KCMC serves primarily as a referral pMTCT centre for these other facilities and provides anti-retroviral prophylactics to HIV-positive pregnant women and their newborns. All pregnant women attending the antenatal clinics were offered VCT. The HIV test result was disclosed on the same day in a one-to-one post-counselling session followed by 'healthy living' information, including infant feeding counselling. HIV-positive women were encouraged to bring their husbands/sexual partners for VCT free of charge.

### Study participants

The study participants were 25 female nurse-counsellors, working at the four pMTCT sites in Moshi town. All nurse-counsellors working at the pMTCT areas in these facilities were eligible to participate and they were informed about the purpose and relevance of the study. Six counsellors were recruited from each of the four sites and from different sections of maternity care within each site including antenatal clinics, labour wards, postnatal and neonatal wards. In addition, the overall supervisor of the pMTCT programmes in Moshi district was included in the study. The recruitment of study participants was based on their availability and willingness to participate. At all facilities, the counselling work was organised on a part-time basis. No full-time counsellors were employed at the time.

The counsellors were given a small sum of money called 'transport allowance' as motivation. The counsellors were all nursing officers holding diplomas in nursing and midwifery; six of them had an additional diploma in public health. Their ages ranged from 26 to 52 years. Only two of the counsellors, including the supervisor, had been trained specifically in HIV and infant feeding counselling, while sixteen had received four weeks of orientation training for general HIV counselling. Eleven had also been trained in breastfeeding counselling in the 1990s during the Baby Friendly Hospital Initiative (BFHI) campaigns. All had counselled mothers on breastfeeding in general and/or HIV-positive mothers on safer infant feeding options. Their experiences in HIV counselling ranged from 1 to 3 years. During the study period each of the four pMTCT sites counselled 7 to 12 women per day.

### Study design and data collection

The study was designed as part of a formative research study aimed at developing locally adapted counselling tools, and was based on fieldwork in the Kilimanjaro region from August 2003 to June 2004. In order to strengthen the credibility of the study findings, a triangulation of methods was used. Twenty-five in-depth interviews and three FGDs with the same study participants (8 participants in each group) were held using semi-structured interview/topic guides. The counsellors' supervisor was purposely excluded during FGDs to allow a free-flowing discussion. The first author of this article (who is a nurse/midwife and a counsellor with a background in sociology and public health, and a native of this area) conducted the interviews. She was assisted by a research assistant during FGDs and she served as a moderator. The interview/topic guides were developed by the research team and were partly adopted from the WHO-recommended sample questions for formative research on HIV and infant feeding [33].

In-depth interviews aimed at eliciting individual perceptions and experiences with infant feeding counselling, while FGDs were to explore collective norms, ideas, experiences and possible divergent views related to their role as infant feeding counsellors. Each interview/discussion built on the previous one with slight modification, elaboration or a better-focused set of themes for discussion. No stratification of the focus groups took place because each participant registered according to the time most convenient personally. While the FGDs were tape-recorded, the individual interviews were recorded in writing. Hence, all interviews were conducted in Swahili, the national language. In addition, the pre-service training curriculum for nurse/midwives was reviewed to investigate how nurse-counsellors were prepared for the role as counsellors in general and as infant feeding counsellors in particular.

### Ethical clearance

Ethical clearance for the study was obtained from Muhimbili University College of Health Sciences (MUCHS), the KCMC Ethical Committee and the Norwegian Committee of Medical Research Ethics. All participants gave their written consents to participate in the study. Nobody refused to participate or withdrew during the study period. In order to ensure confidentiality and anonymity, each participant's name was changed into a number during the interview.

### Data analysis

The FGDs were transcribed and the transcripts along with in-depth interviews were translated from Swahili to English. The transcripts and the interview notes were read several times and any ambiguous or unclear sections of the translation were checked against the original interview written in Swahili. A qualitative software programme 'Open code' assisted in sorting, classifying and coding the data [34]. The data was analysed using content analysis according to the qualitative analytical framework [35], which consisted of the researcher reading and re-reading the texts, manual coding in the margins, synthesising and grouping of data in the relatively exhaustive categories.

## Results

In the following results section, we will discuss issues related to counsellors' perspectives about the recommended infant feeding options for HIV-positive women and their roles as infant feeding counsellors. Thereafter, we will discuss their perceptions about their working conditions and experiences of stress and frustration connected to the counselling work.

### Counsellors' perspectives concerning the recommended infant feeding options for HIV-positive women

#### Breastfeeding

Data from the interviews and FGDs clearly showed that with few exceptions nurse-counsellors did not see breastfeeding as a safe infant feeding option for HIV-positive women. Almost all counsellors stated that from their point of view infant formula was the preferred infant feeding method for HIV-positive women. When they were asked *"What are your opinions about HIV-positive women who breastfeed?" *only the two counsellors who had participated in the national HIV and infant feeding training said that the women were doing the right thing to breastfeed, while 19 said that the women were doing the wrong thing to breastfeed. Four were neutral, saying that it was the woman's choice. Similarly, in response to the question *"What are your opinions about HIV-positive women who do not breastfeed?" *21 said that HIV-positive women did "the right thing" not to breastfeed, while one thought it was an unfortunate decision and three were neutral. Finally, in response to the question "*Do you think there is one best infant feeding method for HIV-positive women?*" 20 out of 25 counsellors replied "yes, infant formula". Two replied exclusive breastfeeding for four to six months, and the remaining three said there was no single best method.

#### Exclusive breastfeeding

One counsellor questioned the feasibility of exclusive breastfeeding on the basis of the customary way that childcare is organised in Chagga communities. The fact that Chagga women customarily do not carry their babies on the back appeared to have negative implications for the feasibility of exclusive breastfeeding. As one counsellor explained:

*"Chagga mothers do not carry their babies on the back when they leave the house like women in the coastal areas do. Babies are usually left with their elder siblings or elderly people like a grandmother, and they are given cow's milk or porridge mixed with cow's milk at a very early age, mostly from two months when the mother is away." *(Interview no. 12; with 2 years pMTCT counselling experience)

Most counsellors during FGDs were concerned that the poor nutritional status of the mother is a major obstacle to exclusive breastfeeding. The following quote illustrates:

"Most women do not have enough food to have sufficient breast milk for the babies after two to three months. It is a waste of time preaching exclusive breastfeeding of a baby at that age – they will mix feed anyway."

While traditionally the confinement period was six months among the Chagga, very few families can afford such a long period of rest after delivery these days. The conditions for exclusive breastfeeding have thus become weaker in the course of modernisation and increasing poverty. This was quoted during FGDs:

"Nowadays most mothers do not stay indoors for more than two months after delivery. They are expected to go out to work so that they can supplement the family income. Life is becoming more and more expensive."

#### Replacement feeding

The counsellors were sceptical about the affordability, feasibility, acceptability and safety of infant formula. They all agreed that it is simply too costly for ordinary people to buy the number of tins necessary to feed their infants in a safe way with infant formula:

"Most families cannot afford to buy their own meals. Where will they get the money for buying formula or cow's milk until the baby is six months of age? A month's supply of formula costs approximately 30,000 Tsh. – almost a minimum wage."

The counsellors explained that the issue is not only one of cost. The practical problems involved in preparing and storing the infant formula makes it an option that is extremely difficult to adhere to exclusively:

"Preparing formula is time-consuming, especially without refrigeration, running water, or an adequate supply of fuel for boiling water. These problems cause many HIV-positive mothers to breastfeed or practise mixed feeding, even if they have access to formula."

The counsellors warned about the problems associated with the storage of formula and cow's milk in a situation where only few people have a refrigerator at home:

"Replacement milk is often kept in a thermos during the day and also at night. This may cause more harm than benefit to the health of babies."

According to the counsellors, not only the storage of the milk, but also the quality of purchased fresh cows' milk may compromise the safety of this feeding method:

"The safety (dilution) of fresh cow's milk is generally questionable unless the family owns a cow because most sellers are not trustworthy any more – they add some water before selling the milk."

Another major threat to both feasibility and acceptability of replacement feeding is connected to disclosure to partner. Mixed feeding in situations of non-disclosure to partner is, according to the counsellors, a likely outcome.

"Formula feeding is easier if the baby's father knows the mother's HIV status and supports her decision. But stigma and secrecy surrounding HIV/AIDS lead most women not to disclose their HIV status."

### Heat-treated breast milk

When it comes to expression and the heat treatment of breast milk the counsellors doubted that the women would be able to express sufficient amounts of milk. More important, however, was their concern that this method would not be acceptable in the community. They explained that the expression of breast milk is highly associated with the death of a child and that it is considered abnormal for a woman with a healthy baby to express her breast milk. One of the counsellors added: *"She becomes a witch – she does not crave for the survival of her child" *(Interview no. 5; with 1 year pMTCT counselling experience)

### Wet-nursing

Counsellors were reluctant to promote wet-nursing, citing an incident where a grandmother purportedly contracted HIV from the grandchild that she was nursing following the death of the child's mother. (There was no evidence, however, that the grandmother was tested prior to initiating wet-nursing). They also commented that very few women in the community know their HIV status and that because of the high HIV prevalence in that area, women fear being tested. Wet-nursing was therefore considered very risky in terms of HIV transmission. Besides being considered unacceptable and unsafe respectively, both expressed, heat treated breast milk and wet-nursing raised serious concerns among the counsellors about the risk of disclosure of the mother's HIV status. One of the counsellors elaborated:

*"I find it difficult to talk about wet-nursing or expression and heat treatment of breast milk. With the rapid spread of HIV knowledge in the community nowadays it will automatically disclose a woman's HIV status." *(Interview no. 22; with 2 years pMTCT counselling experience)

### Study participants' roles as infant feeding counsellors

#### Mothers' expectations

Some counsellors during discussions said they had problems waiting for the patients to decide for themselves what they would do in terms of infant feeding. It was very tempting for many to tell the women what "would be best for them", to give them "the correct answer". The following quote illustrates:

"We are used to instinctively giving advice on health issues and health behaviours. Now counselling is more than this. We are told to let people decide for themselves regardless of whether they are right or wrong. Yet our clients do not understand why we are no longer advising them on what is best for their health. They think we are becoming rude and irresponsible. Their expectations are to get correct answers from us. I'm really in a dilemma and confused. I don't know if I'm doing right to leave my client unsatisfied."

There was a common perception among the counsellors that they, as professional nurses, were supposed to know what would be best for their clients as regards choice of infant feeding method. They said that their clients (women) visiting the pMTCT clinic, expected to get advice and correct answers from the nurses. Now they were worried that their position as knowledgeable professionals was being undermined through their role as pMTCT counsellors. This apprehension of the expectations from the community is reflected in the following comment during discussions:

"When we don't give them a straight answer, they doubt our knowledge, saying nurses do not know much nowadays. We look like fools."

Another issue undermining trust in the nursing profession was, according to the counsellors, that what they were trained to tell the mothers in the pMTCT programme about breastfeeding was very much at odds with the unambiguous messages that they had been trained to teach during the Baby Friendly Hospital Initiative Campaigns.

"It is not very long ago that we were at the frontline advocating for every woman to breastfeed her newborn baby. Now comes another kind of advice – if HIV-positive woman chooses not to breastfeed, we should support that choice. It shows double standards in the care we are giving."

### Lack of confidence and skills in HIV and infant feeding counselling

The nurses complained about lack of confidence in their knowledge of pMTCT. The responses during interviews and FGDs reflected their uncertainty about the medical risks of MTCT and the safety of the different feeding methods. They also attributed this to poor training, out-dated training or no training at all. As one counsellor said:

*"I have been working for more than twenty years as a public health nurse, routinely educating mothers on prevailing health problems. I have only attended one workshop for one week on promoting exclusive breastfeeding. I'm still using the same knowledge to educate mothers on how to feed their babies. I feel like I'm not knowledgeable enough to give my clients updates, especially in this time of AIDS." *(Interview no.23; with almost 3 years pMTCT counselling experience)

The counsellors were concerned that the timing of infant feeding counselling was inappropriate (immediately after a pregnant woman has received her HIV test results). They questioned both the timing and whether a mother would be able to understand or digest any further information. However, the counsellors during discussions perceived this routine as difficult to change since it was part of the pMTCT package decided upon by the hospital management:

"It has been done like this from the beginning of the programme and there is no way we can change it. It was planned by the hospital management and we were not involved."

#### Conflicting loyalties

Many of the counsellors were uncomfortable with the strict confidentiality rules of counselling. In general, they were concerned about the fact that confidentiality aiming to protect the individual woman could work to expose others in her environment to HIV infection as expressed in the following quote:

"If the husband is your own brother, you are not allowed professionally to warn him to take precautions, even when the wife doesn't want to disclose her HIV status to him. I feel bad because this is killing your own brother, and I'm not sure if this is allowed according to the ethics of preventing diseases."

### Working conditions

#### Workload

A recurring theme in interviews and FGDs was that the counsellors felt overwhelmed by a constantly increasing workload. The pressure to compromise the quality of work for the sake of increased workload is expressed in the following quotes:

"The introduction of the pMTCT project in the health facilities has placed an extra load on us because there are many clients waiting to be attended in a very limited time."

"We are working like machines now, it is not possible to stay with one client for long because you have to finish the clients outside, and at the end of the day you need to register how many clients you have attended."

But even though the allowance obtained through counselling is referred to as minimal, it was seen as an important contribution to the family income during FGDs:

"We come here during our days off. We are tired, but because we need this small token called transport allowance to complement the low salary, we have to push ourselves to come, but psychologically and physically we are worn-out from working throughout the week without any rest."

#### Access to information and reference material

The nurse-counsellors reported having very limited opportunities to keep themselves updated. Considering their many competing concerns related to family life, seeking work-related knowledge during time off was not considered a priority. As one counsellor responded during group discussion:

"We have great demands from our own families for survival. I don't think anyone here can get time to go to the library to read after work. We have to look for some extra money to top up our low salaries."

The counsellors also complained about the lack of reference material to help them remember the things they ought to inform the mothers during infant feeding counselling:

"We are overworked, and yet even when you are very tired you are expected to remember all the steps required as written in books. Are we computers that remember everything? We need to have something written down to refer to when counselling mothers."

Lack of tools for demonstrations on how to prepare cow's milk and infant formula was also said to compromise the quality of work as mothers need to see how the preparation should be done to fully understand and remember the procedure.

#### Inability to make home visits

Another issue that was experienced as unsatisfactory by the counsellors was the lack of support to follow up women after they had given birth:

"There is no transport for us to do follow-up of our clients at home. We cannot say anything about the outcome of our work."

"Our counselling work is not complete because we don't know what happens to our clients when they go home after being counselled at the clinic."

### Stress and frustration

#### Hopelessness and death

Many of the counsellors found that they were trapped in a feeling of hopelessness and that their work had little in common with the ambition to heal, which they saw as the very heart of the nursing profession. The following quotes illustrate:

"HIV/AIDS has increased our feeling of hopelessness. We had chosen this profession to heal, but now we have to watch people dying slowly. We have very little to prevent them from dying."

At the same time, the nurse-counsellors were reminded about their own vulnerability in the HIV epidemic. A high level of identification with the patient added to the feeling of hopelessness:

Thinking about the situation at our work, we feel more hopeless and helpless as it always reminds us that, at the end of the day it may be you in that situation of that client, and there is no cure for HIV infection."

Some counsellors expressed signs of depression and burn-out during interviews, and they were aware that this affected the quality of the services they offered:

*"I feel down morally and spiritually when most clients tested on that day are HIV- positive. I get much stressed and I feel very sad deep down in my heart. This feeling distorts all my happiness for that day." *(Interview no. 13; with 1 year pMTCT counselling experience)

*"You get home exhausted, and when you think back at the end of the day you end up frustrated because you did not give adequate care to your clients, is only counting how many clients you have attended in that day. Sometimes we are rude to clients and to our own children because of stress and tiredness." *(Interview no. 2; with 3 years pMTCT counselling experience)

At the same time, some counsellors in the FGDs felt that they were being judged unfairly:

"Like any other human being you become aggressive when you are tired and emotionally distressed. We are like any other human beings, we are always faced with distressed people to whom we have very little to offer, it's frustrating, and it is not fair when people say we are rude."

## Discussion

The present study addressed the well-documented widespread problem of sub-optimal infant feeding counselling in pMTCT programmes in low income settings, and set out to explore this issue from the viewpoint of the counsellors themselves. The following discussion will focus on significant issues related to the counselling work that appeared to be of major importance for the quality of the counselling offered in the pMTCT programmes in Kilimanjaro region.

### Trust

The HIV pandemic has brought about major transitions in terms of nurses' assignments, not least manifested in the major shift in the nursing role from health educators to counsellors. Counselling is a highly complex relational process which requires both knowledge and professional confidence and skills on the part of the counsellor, as well as trust on the part of the client. It requires a very different approach to patient interaction from traditional nursing – an approach that in the present study was found highly challenging to nurses and clients/patients alike [36]. Skills in infant feeding counselling are not yet covered in the nursing curriculum, and the nurses do not feel that they have sufficient competence in their new roles as counsellors.

Moreover, nurses experience that their roles as educated individuals with particular trusted skills and knowledge have become threatened by their newly gained roles as counsellors operating within an atmosphere of patient self-determination and health-related decisions resting with the patient. According to the nurses in the study, on their part the pMTCT clients do not feel comfortable with the newly gained roles of the nurses either. Patients expect to be told what is right and wrong and what they should do to prevent illness or to heal disease, and they feel betrayed by nurses who appear to lack the necessary authoritative knowledge that can help them. Both nurses and clients feel that the counselling role leaves nurses with a diffuse guiding role, a role that is vague to the extent that it generates a substantial problem of trust. Indeed, in the case of pMTCT, the challenge of trust is perceived as threatening the very confidence and faith that clients or patients have customarily had in nurses.

The problem of trust should also be viewed in the light of the knowledge on which pMTCT rests. In the case of infant feeding counselling in pMTCT programmes, knowledge of how to reduce HIV transmission through breastfeeding is vested in the counsellors. A major counselling dilemma as documented in this study is that most counsellors believed that formula feeding was the 'right way' for an HIV-positive woman to feed her infant. The implications of this perception may however be fatal to the lives of babies in a context where most HIV-positive women are too poor to practice safe replacement feeding. This finding is contrary to the previous findings of a study conducted in the same area by de Paoli and colleagues [32], which documented that the counsellors distrusted replacement feeding and were inclined to advise HIV-positive women to breastfeed. This difference might be explained by the increased public attention given to pMTCT and HIV transmission through breastfeeding during recent years.

A basic condition for successful pMTCT counselling is that the counsellor not only has confidence in her own professional knowledge, but also in the relevance and applicability of this knowledge for the individual woman in her particular situation. The findings in this study show that the nurse-counsellors do not have this kind of confidence in the work they are set to do. Nurse-counsellors would continuously state that they were not well enough informed or skilled about MTCT to be able to present the message well enough for the mothers to make 'informed choices'. What appears as more serious however, is that the nurses in the study simply did not believe that any of the alternative infant feeding methods they were proposing to the mothers – including exclusive breastfeeding, cow's milk feeding or formula feeding – were either socio-culturally acceptable or practically feasible in the social and cultural context of the Kilimanjaro region. Wet-nursing and the expression and heat treatment of breast milk emerged as so farfetched in the present context that they were not introduced as options for the mothers to consider. At none of the research sites did the nurse-counsellors believe that most of the mothers would be able to adhere to either exclusive breast feeding, formula feeding or other replacement feeding, as these methods violated cultural norms or were too impractical. Consequently the nurses simply did not believe in the very health-promoting concept they were set to work with.

### Motivation

The experience of job motivation and job satisfaction is closely linked to the experience of doing an important and meaningful job. Lack of trust in both the role as nurse-counsellor and in the measures proposed to prevent mother-to-child transmission was experienced as highly damaging for the motivation of the work as a nurse. The lack of motivation for and confidence in the work as pMTCT counsellor was encountered in contexts characterised by severe shortage of staff and immense time constraints that left the nurse with merely a few minutes to present and discuss the complex pros and cons of the various infant feeding options with each client. The clients were women who had just received an HIV-positive diagnosis and who had an enormous demand for nursing care and for someone to talk to. The time constraint thus emerged in this context as inhuman and was challenging the very core of nursing care. The combined challenges experienced by the pMTCT counsellors generated immense frustration and an experience of job-related meaninglessness. This is also in line with findings from a study in South Africa by Buskens and colleagues [23,24].

### Global policies in local context

The dynamics in the encounter between highly complex and biomedically founded pMTCT regimes and the realities of local African women's lives proved to be challenging to the extent that it caused confusion for nurses and clients alike. Several studies have documented the key role of nurses and midwives in influencing mothers' positive decisions on infant feeding [16,17]. Other studies have documented that, with formal and supportive supervision, nurses can significantly increase the rates of exclusive breastfeeding [18-20]. This study indicates that in the context of the present pMTCT initiatives in the Kilimanjaro region there appears to be a long way to go before similar positive results can be recorded. Based on the challenges encountered by nurse-counsellors in the present pMTCT programme combined with the problems that mothers face trying to adhere to the recommended feeding methods [37], the impact of the infant feeding component of the pMTCT programme on infant feeding outcomes is uncertain.

### Limitations

In interpreting the findings of the present study, several limitations must be acknowledged. The relatively small number of pMTCT nurse-counsellors participating in this study may not be representative of the nurse-counsellors working in the Kilimanjaro region and even less in Tanzania as a whole. We do believe however, that the results which are based not on one, but on four pMTCT programmes in Moshi, and which are collected through a triangulation of research methods, have considerable relevance for pMTCT programmes well beyond the four study sites. Furthermore, the scope of the study is limited. A more comprehensive exploration of problems that compromise the quality of counselling would involve other groups of study participants – primarily HIV-positive women (for their views on counselling services) and hospital administrators (for structural issues). These groups of study participants are however included in a forthcoming publication.

## Conclusion

In this paper, we have explored the experiences of nurse-counsellors responsible for counselling HIV-positive women on infant feeding in pMTCT programmes. We conclude that the experiences of the study participants were characterised by combined challenges related to the shift from a health-educator to a nurse-counsellor role and the enormous work burden, as well as a fundamental lack of confidence in the feasibility of the infant feeding component of the pMTCT programme in this local African context. One important question that emerged is: *how can nurse-counsellors implement the proper promotion of a component package they do not believe in? *The paper supports the critical notion that successful counselling is hardly a matter of biomedical or nursing knowledge and practice alone. Counselling, even more than traditional nursing, requires time and a fundamental knowledge of the socio-cultural environments within which particular health-related issues are addressed.

In light of the above findings, the conditions under which nurse-counsellors are expected to provide good quality counselling services are critically questioned. To improve these conditions and the confidence of counsellors, infant feeding counselling training and skills development as reflected in the policy guidelines is fundamental and should be integrated into pre-service and in-service training courses. Furthermore, culturally-appropriate counselling tools can be developed as a way to improve the standardisation and routine of infant feeding counselling. However, though important, elevating the level of knowledge, skills and confidence of the nurse-counsellors does not address the fundamental issue of the acceptability and feasibility of the infant feeding methods in the local community. Community-based approaches to increasing the acceptability of the safer infant feeding options – and in particular exclusive breastfeeding – should be strengthened. At the same time continuing research aiming to improve the safety, feasibility and acceptability of the recommended infant feeding methods for HIV-positive mothers is urgently needed.

## Competing interests

The author(s) declare that they have no competing interests.

## Authors' contributions

SCL contributed to the conception and design of the study, conducted the data collection, and was responsible for the analysis of the data. She drafted the manuscript and revised it. AB and KMM contributed to the conception and design of the study. AB, MDP and KMM critically reviewed draft versions of the manuscript. All authors read and approved the final version of this manuscript.
